# The Carbon-Nitrogen Balance of the Nodule and Its Regulation under Elevated Carbon Dioxide Concentration

**DOI:** 10.1155/2014/507946

**Published:** 2014-05-28

**Authors:** Marc Libault

**Affiliations:** Department of Microbiology and Plant Biology, University of Oklahoma, 770 Van Vleet Oval, Norman, OK 73019, USA

## Abstract

Legumes have developed a unique way to interact with bacteria: in addition to preventing infection from pathogenic bacteria like any other plant, legumes also developed a mutualistic symbiotic relationship with one gender of soil bacteria: rhizobium. This interaction leads to the development of a new root organ, the nodule, where the differentiated bacteria fix for the plant the atmospheric dinitrogen (atmN_2_). In exchange, the symbiont will benefit from a permanent source of carbon compounds, products of the photosynthesis. The substantial amounts of fixed carbon dioxide dedicated to the symbiont imposed to the plant a tight regulation of the nodulation process to balance carbon and nitrogen incomes and outcomes. Climate change including the increase of the concentration of the atmospheric carbon dioxide is going to modify the rates of plant photosynthesis, the balance between nitrogen and carbon, and, as a consequence, the regulatory mechanisms of the nodulation process. This review focuses on the regulatory mechanisms controlling carbon/nitrogen balances in the context of legume nodulation and discusses how the change in atmospheric carbon dioxide concentration could affect nodulation efficiency.

## 1. Introduction

Plant-bacteria interactions are diverse in nature. While bacterial infections of plant cells are mostly perceived as pathogenic and lead to the activation of the plant defense system, some could lead to commensalism or, as described mostly in legumes, to mutualistic symbiotic interactions. Nodulation, with mycorrhization, is one of the best studied mutualistic symbiotic interactions between plant and microorganisms. Nodulation is the product of a controlled infection process of the legume root system by soil bacteria of genus* Rhizobia* and results in the development of a new plant root organ, the nodule, where differentiated bacteria named bacteroides fix and assimilate for the plant the atmospheric dinitrogen.

Because bacteria invest a lot of energy in fixing atmN_2_ (atmN_2_ + 8e^−^ + 8H^+^ + 16ATP = 2NH_3_ + H_2_ + 16ADP + 16P_*i*_) and because legumes provide to the bacteroides a significant amount of photosynthates (5 to 10 grams of carbons per one gram of fixed nitrogen [1]), the nodulation process is a high cost biological process for both partners. Hence, one critical aspect of the nodulation process is the establishment of well-balanced interactions between the two partners to lead to beneficial outcomes for both organisms. This interaction is highly dependent on communication between the two partners before, during, and after the initial infection process.

The molecular mechanisms controlling the recognition of the two partners, the initial infection of legume root hair cells by mutualistic symbiotic bacteria (i.e., root hair curling, invasion of the root hair cell by symbiotic nitrogen-fixing bacteria through the development, and elongation of the infection thread), legume nodule organogenesis, and the role of plant hormones in controlling nodulation have all been investigated during the past 15 years using forward and reverse genetic tools. These studies have been extensively documented and reviewed [2–7]. Similar strategies combined with elegant grafting and split-root experiments have be utilized to characterize the legumes genes controlling the autoregulation of legume nodulation (e.g., signal exchanges between the shoot and the root systems) [8–11]. For example, in the context of mutant shoot, where the shoot-root communication is jeopardized, nodulation is strongly enhanced leading to a hypernodulation phenotype [12–16]. Interestingly, plants showing a hypernodulation phenotype do not have an enhanced uptake of atmN_2_ compared to wild type plants [17, 18]. This latter result strongly supports that additional molecular mechanisms are controlling atmN_2_ fixation independently of the infection level of the legume plant by symbiotic bacteria and nodule development. This review summarizes the cellular and molecular mechanisms regulating the interactions between infected plant cells and rhizobia and discusses the potential effects of the increase of the concentration of atmospheric carbon dioxide on these interactions.

## 2. The Secret to This Long-Term Relationship: The Selection of the Right Symbiotic Partners

Nodules and more specifically the bacteroides are considered as an important sink of plant photosynthates. Consequently, a successful nodulation relies on controlling the exchange of nutrients between the plant and the bacteria. This clearly delimits plant-microbe symbiotic to pathogenic relationships. Therefore, the coevolution between legumes and symbiotic bacteria is highly dependent on photosynthetic and symbiotic performances of the two partners (i.e., atmN_2_ fixation efficiency; photosynthesis activity). Based on this concept, and to face the disparity of the photosynthesis activity among various legume cultivars, many studies identify symbiotic strains characterized by various atmN_2_ fixation efficiencies [19–22].

The diversity of the microbial community in soil is leading to the presence of multiple bacterial lineages which can simultaneously infect the same legume plant. This competition for nodulation leads to the development of a heterogeneous pool of low- and high-efficiency atmN_2_-fixation nodules [23–25]. Not surprisingly, the infection of the plant by less effective rhizobia strains which are characterized by low efficiency in fixing atmN_2_ and high uptake of plant photosynthates is major limitation to plant development. To select their energy preferred symbionts and maximize nitrogen uptake without drastically affecting plant carbon resources, the plant developed various cellular and molecular mechanisms such as the promotion of the infection of the root hair cells and nodule by one single bacteria strain. In addition, ecological and physiological approaches have clearly demonstrated that legumes can monitor and respond to the nitrogen-fixing performance of symbiotic bacteria [26], punishing their low-efficiency hosts by reducing rhizobial viability and, ultimately, promoting nodule degeneration [26–29].

These are contributing factors to better discriminate and “punish” the low versus highly efficient atmN_2_-fixing bacteria. Ultimately, the repetitive selection of the favorite symbionts by the host will affect the microbial ecosystem: after plant death and nodule degeneration, a significant population of the most successful symbiotic rhizobia will be released in the rhizosphere increasing their representation compared to low efficient rhizobia, bacteria strains slowly growing in the soil due to limited nutrient availability. Hence, by preventing infection by nonfixing rhizobia and increasing the overall population of highly efficient strains in the rhizosphere, one long-term outcome of the legume-rhizobia symbiosis is the preferential selection of the most beneficial bacteria strains by the plant to maximize nitrogen fixation. This concept of sanctions by the plant hosts against low efficient bacteria to maximize atmN_2_ fixation is supported by mathematical model [30].

The preferential selection of specific rhizobia strains is likely a major reason supporting the conservation of the plant and bacteria genes required for mutualism [31]. One visible result of the coevolution of host plant and bacterial strains is the preferential concentration of* R. etli* carrying the* nodC* allele type-*α* and type-*δ* in the Mesoamerican and Andean soils, respectively [32]. Recently, the characterization of the molecular mechanisms controlling the preferential colonization of legume roots by highly efficient rhizobia strains has been initiated. Zanetti et al. [33] identified* P. vulgaris NF-YC1*, a gene encoding a C subunit of the heterotrimeric nuclear factor NF-Y transcription factor [34] as a key regulator of the infection of the plant by the most efficient strains of rhizobia. In their analysis, Zanetti et al. [33] characterized the putative orthologs of Pv*NF-YC1* in various plant species including* Arabidopsis thaliana* (At1g08970, At*NF-YC9*) and* Glycine max* (Glyma19g42460). In* M. truncatula*, two genes (Medtr1g082660; Medtr7g113680) are syntenic to Glyma19g42460. Upon mining of the soybean and medicago transcriptome atlases [35–39] and similarly to Pv*NF-YC1* [33], Glyma19g42460, Medtr1g082660 and Medtr7g113680 are ubiquitously expressed and not transcriptionally regulated in response to rhizobia (see Supplementary Figure 1 available online at http://dx.doi.org/10.1155/2014/507946). The ubiquitous expression pattern of these* NF-YC* legume genes suggests they control biological functions other than legume nodulation (e.g., At*NF-YC9*, Pv*NF-YC1* orthologous gene, controls* A. thaliana* floral transition [40]). It is likely possible that the regulation of legume nodulation by NF-YC proteins is dependent on their interaction with nodulation-specific A and B NF-Y subunits of the heterotrimeric CAATT transcription factor.

## 3. Cellular Communication between the Infected Plant Cells and the Bacteroides

While legume nodulation is initiated by the infection of the plant host by selected rhizobia, the long-term outcome the legume nodulation is the establishment of the symbiosis between the two partners. To reach this goal, a permanent communication between the plant cells (i.e., root hair cells and infected cells of the nodule) and the symbiosome is required. This organelle-like results of the endocytosis of bacteroides in the infected plant cell. It is delimited by the symbiosome membrane (SymM) and contains a limited number of bacteroides separated one another by the symbiosome space (SymS) [41] (usually two to four in determinate infected cells, only one in indeterminate infected cells [42–44]).

Transporters located in the SymM are playing essential roles in balancing the fluxes of metabolites between the plant cell and the bacteroides (see below). Very interestingly, the proteome of the symbiosome very nicely reflects the complexity of the interaction existing between the plant host and the symbiotic bacteria [45]. In fact, several molecular studies clearly highlight the complex molecular organization of the symbiosome based on the translocation of plant proteins into the symbiosome membrane or cytoplasm or both. Plant protein translocation to the SymS depends on the presence of peptidic symbiosome-localization sequences [46, 47]. Lending even more support to the impact of plant proteins in regulating symbiont biology, nodule-specific cysteine-rich (NCR) peptides synthetized by the galegoid legume cells were demonstrated to be essential to bacterial endoreplication, a cellular process characteristic of fully differentiated bacteroides [48–51]. In* M. truncatula*, NCR peptides are directed to the symbiosomes by the defective in nitrogen fixation 1 (DNF1) protein, a component of the signal peptidase complex [52]. Ultimately, the NCR peptides will traffic to the bacterial periplasm and/or cytoplasm.

The role of these various plant proteins in regulating bacterial endoreplication clearly highlights the influence of the plant host on the symbiont. This likely allows the plant to exhibit better control of bacteroid differentiation and, as a result, exhibit better control of nitrogen fixation. To better support the relationship existing between the plant, the bacterium, and nitrogen fixation, transcriptomic and physiological experiments on* M. truncatula* mature nodules clearly show a decrease of the expression of more than 120 NCR genes and the decrease of nitrogenase activity upon nitrate treatment [53]. In addition to their role in bacteroid endoreplication, plant NCR proteins also act as antimicrobial chemical [54]. Adapting to the presence of these peptides, bacteroides synthetize the BacA protein, an ATP-binding cassette superfamily (ABC) [54–56]. Together, these studies support a complex interaction between plant and bacteria during the latter stage of nodulation where both partners developed specific sets of genes allowing controlling the nodulation process.

## 4. Transport, Conversion, and Storage of the Products of Plant Photosynthesis during Legume Nodulation

Plant cell-rhizobia symbiosis is primarily based on the exchange of metabolites especially sucrose, asparagine, and glutamine, allowing the balanced exchange of nitrogen and carbon. Numerous studies focused on the role of transporters and receptors located in symbiotic biological membranes [57–59]. Balanced carbon and nitrogen exchanges are also dependent on the transport of plant photosynthates to the nodule. There, the products of the photosynthesis are converted into malate in the plant cell through the glycolysis pathway, the Krebs cycle, the phosphoenolpyruvate carboxylase glycolysis, and the intensive nodule CO_2_ dark fixation [60, 61]. Malate is assimilated and used by the symbiont. Ultimately, after its transportation through the SymM [57, 58], carbons will be stored in the bacteroid in the form of poly-3-hydroxybutyrate (PHB) particles. This carbon source can be remobilized by the bacteria in response to a stress (e.g., release of bacteria into the rhizosphere consecutively to nodule degeneration) and is also used as a redox potential to allow bacteroides survival in the anaerobic conditions existing in the nodule, which is the environment necessary for the optimal fixation of atmN_2_ [62].

To better control the uptake of plant photosynthates by the bacteroides, legumes develop molecular strategies to control carbon sequestration in the bacteroides. For example, in addition to regulating nodulation efficiency the soybean gene* Nucleolar*/*Mitochondrial protein involved in Nodulation a* (Gm*NMNa*), initially characterized based on its strong and specific expression in root hair cell and nodules in response to* B. japonicum* inoculation [63, 64], controls bacteroid number in the nodule infected cell as well as the density of PHB granules in bacteroides. The mitochondrial localization of GmNMNa protein and the limited accumulation of PHB in bacteroides upon silencing of Gm*NMNa* support that Gm*NMNa* influences carbon metabolism in infected soybean nodule cells. Previous reports clearly demonstrated the essential role of mitochondria in legume infected cells to presumably maintain plant cell function under microaerobic conditions [65–67].

Interestingly, it seems that the* NMN* gene family is specialized in regulating mutualistic symbiotic plant microbe interactions since the* M. truncatula* gene ortholog to Gm*NMNa* is not only overexpressed during nodulation but also during mycorrhization (Supplementary Figure 2). This latter result suggests the functional redundancy between nodulation and mycorrhization in addition to the conservation of the initial signaling cascade activated in response to the Nod and Myc factors [6].

The essential role of PHB during the nodulation process is also demonstrated by two independent studies. First, the* Sinorhizobium meliloti phbC* mutant, mutant defective in PHB biosynthesis, does not fix atmN_2_ [68]. Second,* Mimosa pudica* nodules infected by a modified strain of the pathogenic* Ralstonia solanacearum* accumulate PHB. This bacterial strain carries a mutation in the* HRPG* gene, gene previously described as a key regulator of bacterial virulence via the type III secretion system (T3SS; [69]), and is expressing the symbiosis genes of* Cupriavidus taiwanensis*, a* M. pudica* symbiotic bacterium [70]. This latter result suggests at least some similarities between rhizobia and pathogenic bacteria in uptaking and storing plant photosynthates. These studies lend even more support that nodulation is the result of a highly controlled interaction between legumes and soil bacteria.

## 5. Evolutionary Perspective of Legume Nodulation in the Context of the Environmental Changes

Legume nodulation is a complex biological process involving multiple levels of coevolution existing between the plant and symbiotic bacteria (i.e., recognition of plant flavonoids by the free-living bacteria, recognition of the bacterial Nod factor by plant receptors lysine kinases, control of bacteria differentiation and endoreplication by plant cells, and balanced exchanges of nutrients between the two organisms). The nodulation process is restricted not only to our knowledge of the infection of the root hair cell by the symbiont but also, mostly, to the establishment of a controlled interaction between the plant and the bacteria. This interaction might be jeopardized due to rapid environmental changes.

The most pessimistic predictions of climate change suggest an increase by 4°C of the current temperature as reported by the Intergovernmental Panel on Climate Change [71] and an atmospheric carbon dioxide (atm[CO_2_]) concentration rising above 800 parts per million by the year 2100 in contrast to 390 ppm today (http://www.ipcc-data.org/observ/ddc_co2.html). Associated with unpredictable rainfall patterns and poor soil management, these environmental changes will affect not only plant growth but also the composition of soil microbial communities including rhizobial communities. Together, the modification of various climatic factors will require an adaptation of the legume nodulation process.

**Figure 1:**
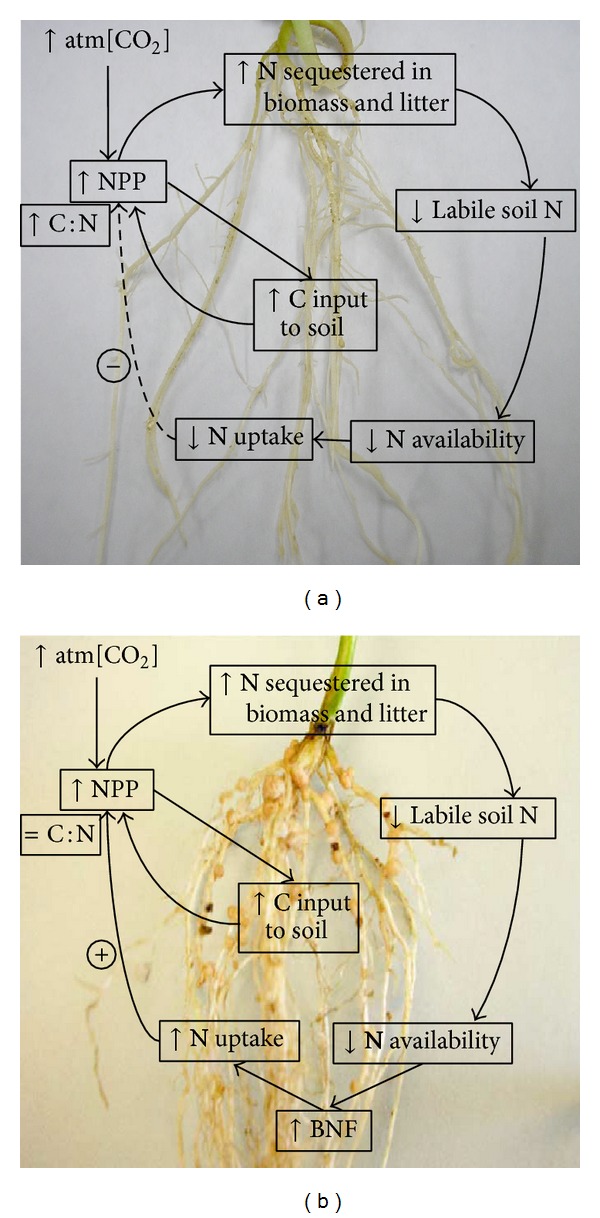
The increase of the concentration of atmospheric carbon dioxide will impact nitrogen uptake by the plants to balance ion balances. On the short term, this massive uptake of nitrogen will lead to the depletion of usable nitrogen resources in soil and the enrichment of the rhizosphere in carbon (upper cycles in both (a) and (b)). As a consequence, the limited access to nitrogen will lead to the unbalance between carbon and nitrogen and, as a consequence, to the limited growth of plants. In the case of legumes (b), limited nitrogen availability will enhance nodulation. The biological fixation of the atmospheric dinitrogen associated with the uptake of carbon dioxide will positively impact the net primary production of the plant (NPP). This figure was adapted from [78].

Among these factors, and based on the tight interactions between the nitrogen and carbon cycles, an increase of the concentration of atm[CO_2_] is going to directly affect the crop's nitrogen/carbon balance and nodulation. To respect the balances between ions, it is predicted that plants may become nutrient limited, including nitrogen-limited, in the context of a greater carbon input [72, 73]. Models support that legumes will overcome the problem of nitrogen limitation by promoting physiological adaptations. These adaptations include the increase in nodule size, the increase in nodule number per plant (potentially correlated with an increase of the number of successful root hair cell infection), and the increase of nitrogenase activity [74–76] (Figure 1). The latter also support the idea of an increase in the allocation of plant photosynthates to the bacteroides and, more globally, of various ions to respect balances. Hence, it is assumed that legumes represent a potential solution to increase carbon sequestration and help to mitigate the impact of atm[CO_2_]. While scientists have clearly demonstrated the impact of atm[CO_2_] on nodulation [60, 77], the molecular mechanisms regulating nodulation under high atm[CO_2_] remain unknown. In addition, it is unclear how the* rhizobia* community will adapt to higher atm[CO_2_] in particular and climate change in general. The study of the adaptation of the microbe and plant as well as their interaction in response to environmental changes represents new avenues of research which could impact food production and sustainability on the long term.

## Supplementary Material

Expression pattern of medicago genes orthologous to the soybean Gm*NMNa*gene (Supplemental Figure 1) and soybean and medicago genes orthologous to the common bean Pv*NF-YC1* (Supplemental Figure 1). Gm*NMNa* and Pv*NF-YC1* are two legume genes controlling the nodulation process.
