# Supplementary material for: The Carbon-Nitrogen Balance of the Nodule and Its Regulation under Elevated Carbon Dioxide Concentration
Source: Biomed Res Int. 2014 May 28;2014:507946. doi: 10.1155/2014/507946 (PMC4058508; doi:10.1155/2014/507946)

Supplemental Figure 1. Expression levels of Medtr1g082660 (blue) and Medtr7g113680 (red), the two medicago genes orthologous to PvNF-YC1, a key regulator of the infection of the legumes by rhizobia. The various conditions, tissues and treatments of the plants used for transcriptomic analysis are indicated on the x-axis. The relative expression of the gene is indicated on the y-axis. The data, were mined from the *Medicago truncatula* gene expression atlas (http://mtgea.noble.org/v3/; Benedito et al., 2008; He et al., 2009), reveal that Medtr1g082660) and Medtr7g113680 are ubiquitously expressed genes; not induced in response to rhizobia inoculation.

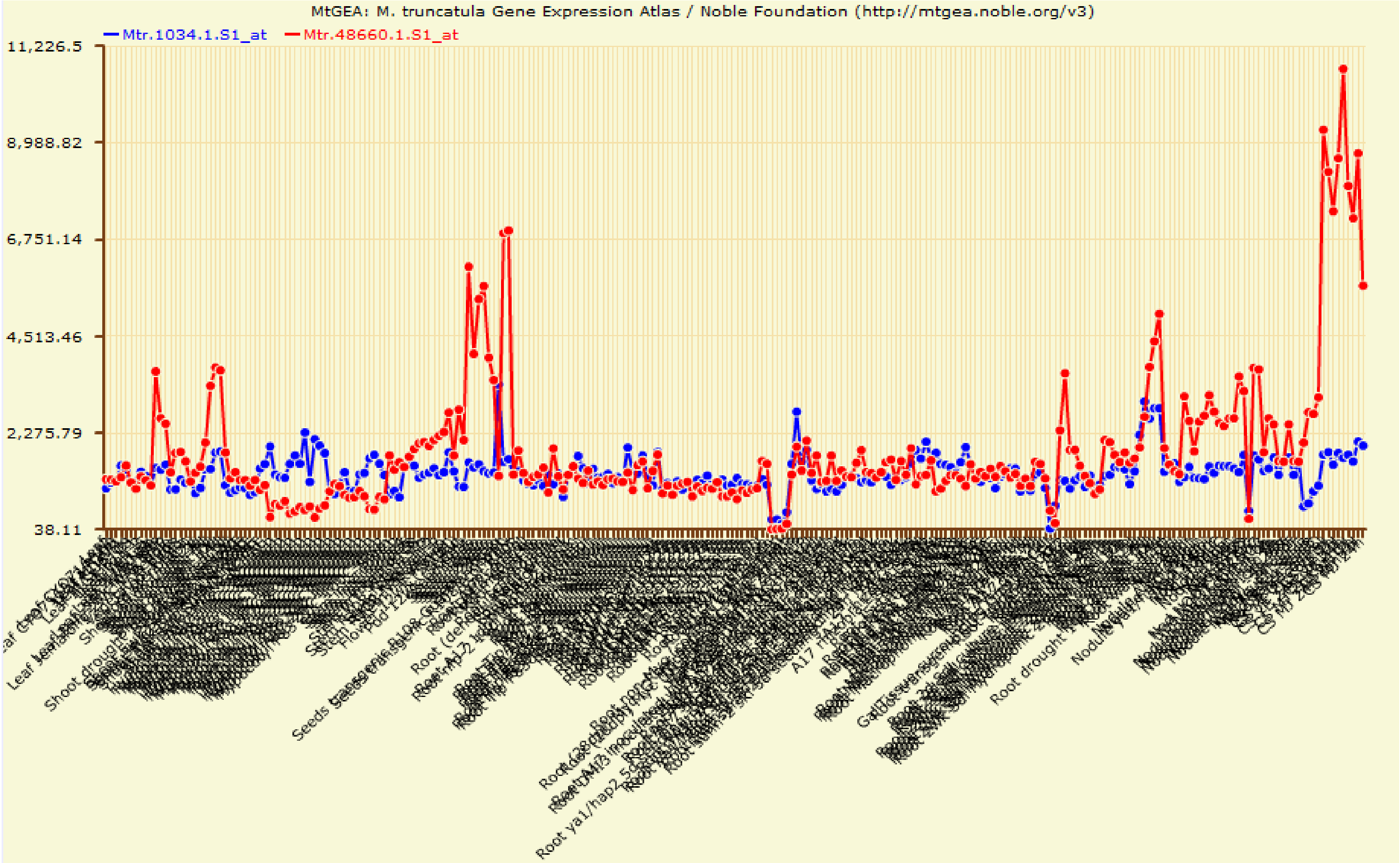

Expression levels (y-axis) of Glyma19g42460, the soybean gene orthologous to PvNF-YC1, in various soybean tissues and at different stages of the nodulation process (x-axis) . The data were mined from two different studies (Libault et al.,2010; Severin at al., 2010) and the figure was generated using Soykb database (http://soykb.org/; Joshi et al., 2012).

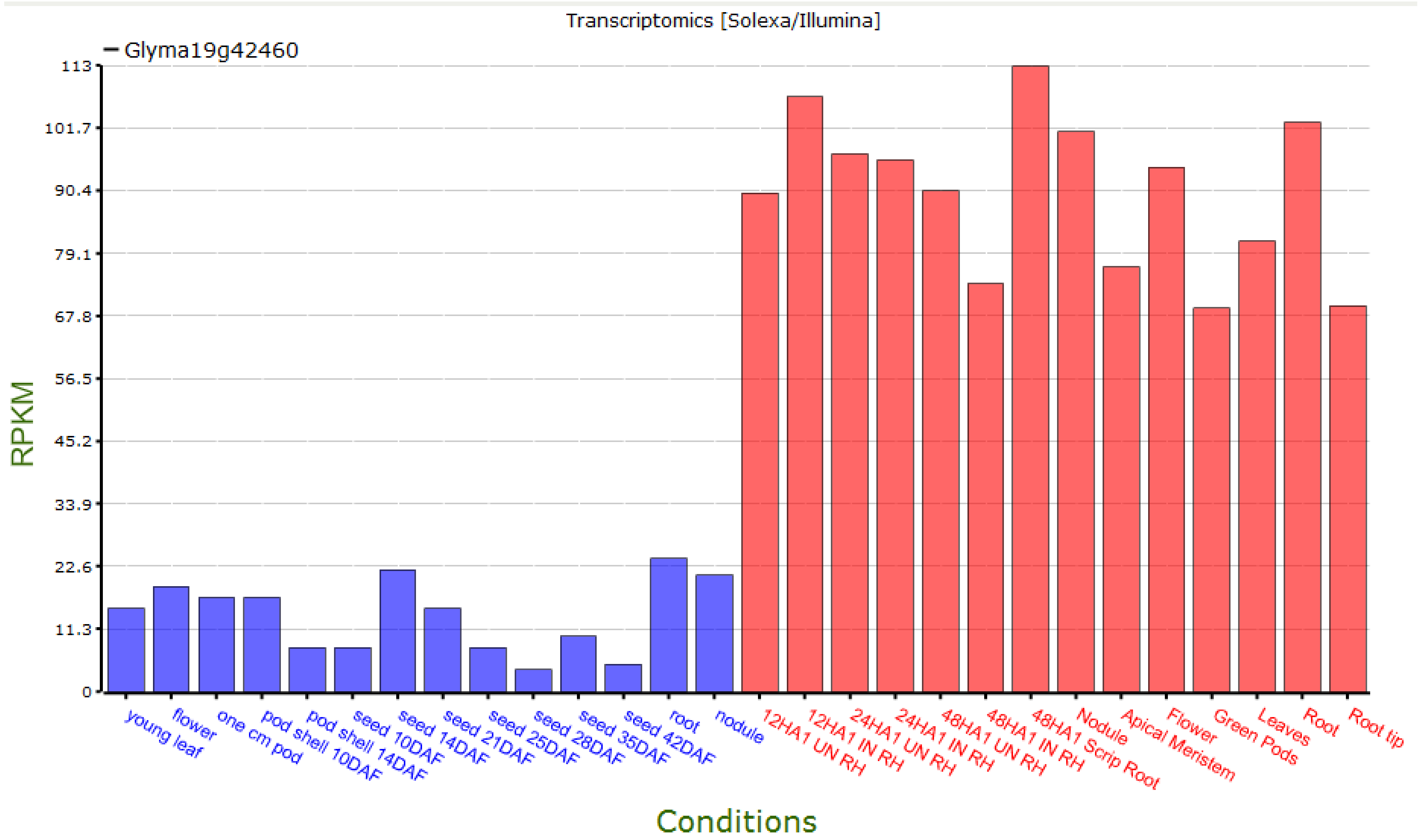

Supplemental Figure 2. Expression levels of Medtr5g005290, the medicago orthologs of Gm*NMNa*. Similarly to Supplemental Figure 1, the various conditions, tissues and treatments of the plants used for transcriptomic analysis are indicated on the x-axis. The relative expression of the gene is indicated on the y-axis. The data were mined from the *Medicago truncatula* gene expression atlas (http://mtgea.noble.org/v3/; Benedito et al., 2008; He et al., 2009). Interestingly, Mt*NMNa* is mostly expressed in response to *Sinorhizobium meliloti* and Nod factor treatments (black arrow) but is also induced in response to mycorrhiza and Myc-LCO (grey arrow).

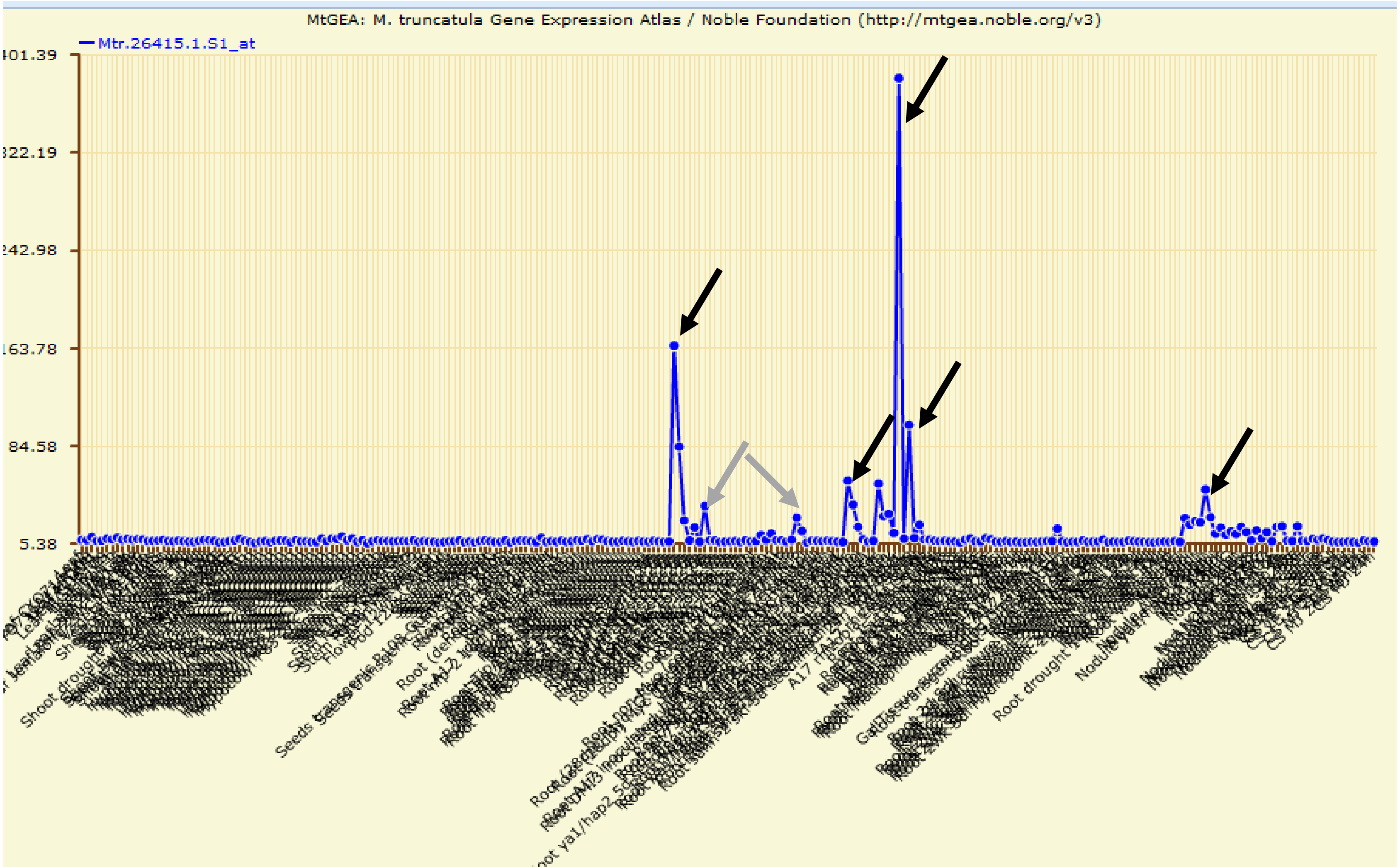

Supplement: Supplementary file 1 — Expression pattern of medicago genes orthologous to the soybean GmNMNagene (Supplemental Figure 1) and soybean and medicago genes orthologous to the common bean PvNF-YC1 (Supplemental Figure 1). GmNMNa and PvNF-YC1 are two legume genes controlling the nodulation process. [file 507946.f1.pdf]
